# Role of CD123 (+) Plasmacytoid Dendritic Cells in Etiologically Different Variants of Erythema Multiforme: A Monocentric Retrospective Study

**DOI:** 10.3390/dermatopathology8020014

**Published:** 2021-04-03

**Authors:** Hatice B. Zengin, Tatsiana Pukhalskaya, Bruce R. Smoller

**Affiliations:** Department of Pathology and Laboratory Medicine, University of Rochester Medical Center (URMC), Rochester, NY 14642, USA; tatsiana_pukhalskaya@urmc.rochester.edu (T.P.); bruce_smoller@urmc.rochester.edu (B.R.S.)

**Keywords:** erythema multiforme, plasmacytoid dendritic cells, CD123

## Abstract

Plasmacytoid dendritic cells (pDCs) constitute a subset of dendritic cells known to be the “professional” interferon type I (IFN-I) producers. pDCs play an important role in antiviral immunity, as well as linking innate and adaptive immunity. Under normal conditions pDCs are not present in skin. They are shown to be a part of the inflammatory infiltrate in different skin conditions including erythema multiforme (EM). This condition is considered to be a cell-mediated immune reaction to a wide variety of agents, most commonly herpes simplex virus. Nevertheless, the pathophysiology of EM still remains unclear. In this study, we grouped 32 biopsies from 30 patients diagnosed with EM, based on their etiology and analyzed the density and distribution of CD123 positive pDCs. In all cases we observed a greatly increased number of pDCs in the dermal inflammatory infiltrate. Virally-induced EM (by herpes simplex virus (HSV) and other viruses) was more likely to have a significantly higher number of pDCs compared to non-virally associated EM. Hence, we think that pDCs play a key role in the pathogenesis of EM independent of etiology and may play an increased role in virally-associated cases. Further studies on pDCs would clarify their importance in EM and improve our understanding of the pathophysiology of this disease.

## 1. Introduction

Plasmacytoid dendritic cells (pDCs) are a unique subset of conventional dendritic cells [1,2,3]. They have an ability to sense viral and bacterial pathogens as well as “self” DNA via the Toll-like receptor [1,2,3]. Under normal conditions they are not present in the skin, but upon cutaneous injury pDCs migrate from the peripheral blood into the affected area [2,3]. They subsequently respond to stimuli through Toll-like receptors 7 and 9 (TLR7 and TLR9) by releasing high quantities of type I interferon, particularly IFN-α, contributing to ongoing inflammatory invents [2,3]. pDCs play a specialized role in antiviral immunity as well as systemic autoimmunity [4]. These cells are commonly detected by their CD123 marker and are described in psoriasis, lupus erythematosus (LE), lichen planus (LP), and cutaneous tumors [3].

Although it is sensible that pDCs might be involved in pathology of erythema multiforme (EM), there is only scattered information about their role in this skin condition [5]. EM often manifests as target-like skin lesions with or without erythematous mucosal lesions [6]. Pathologic findings in EM typically include superficial, perivascular lymphocytic inflammation, basal cell vacuolar degeneration, scattered necrotic keratinocytes, and lymphocyte exocytosis [6]. In general, EM appears to result from a cell-mediated immune reaction to variety of agents i.e., viral (herpes simplex virus), bacterial (mycoplasma pneumonia), drugs, malignancies, and so on [7,8]. Predominance of CD4+ T-lymphocytes and IFN-γ was shown in herpes associated erythema multiforme (HAEM), while in a drug-induced erythema multiforme (DIEM) there is involvement of monocytes, CD8+ T-lymphocytes and tumor necrosis factor-α (TNFα) [9]. Based on these findings, researchers suggested that HAEM and DIEM may result from different underlying pathophysiologies, though it is still not completely clear [9]. We propose that pDCs might be one of the key cells in this matter of divergent pathogeneses. Therefore, the aim of this study was to investigate the density and distribution of plasmacytoid dendritic cells in etiologically different variants of erythema multiforme. Our goal is to advance our understanding of pDCs and their role in the pathophysiology of this cutaneous condition.

## 2. Materials and Methods

We performed an institutional review board-approved retrospective study on archived tissue. Surgical pathology specimens diagnosed with EM between the dates of 1 January 2011 and 28 October 2020 were selected for the analysis. All selected cases were diagnosed by the dermatopathologists in our institution. Patient charts were also reviewed by the authors for clinical information (age, gender, severity), diagnostic confirmation and etiology of EM. On chart review, we categorized the cases with both skin and mucosal surface involvement (EM major) as severe, while the cases with skin involvement only (EM minor) as mild disease. A total of 32 biopsies from 30 patients were included. Two patients were biopsied twice from different body sites. The included cases were divided into 6 categories: herpes simplex virus (HSV)-associated EM (9); mycoplasma pneumonia-induced EM (5); other viral infectious EMs (4); drug induced EM (3); idiopathic (1); and unknown etiology (10). The idiopathic group includes cases in which the etiology was not identified despite extensive work up for common EM culprits, and the cases were accepted and treated as idiopathic by the dermatologist. On the other hand, unknown etiology group includes the cases that were not worked up for etiology. For statistical purposes it is important to add that the recurrent biopsies from the same patients appeared to be in the unknown etiology category.

Immunohistochemical (IHC) studies were performed on 4 µm sections of formalin-fixed, paraffin-embedded tissue using the Leica Bond III instrument. IHC assay consisted of CD123 Leica clone BR4MS in a dilution of 1:100. After incubation for 15 min, the epitope retrieval was performed using high pH antigen retrieval for 30 min. Tissue staining was performed on a Leica BOND III immunostainer with a Leica Refine Polymer Detection Kit. A CD123 stained lymph node was used as a positive control. A biopsy of healthy skin was also stained with CD123 and used as a negative control (no photo included). Rare perivascular CD123 positive cells were noted.

**Figure 1:**
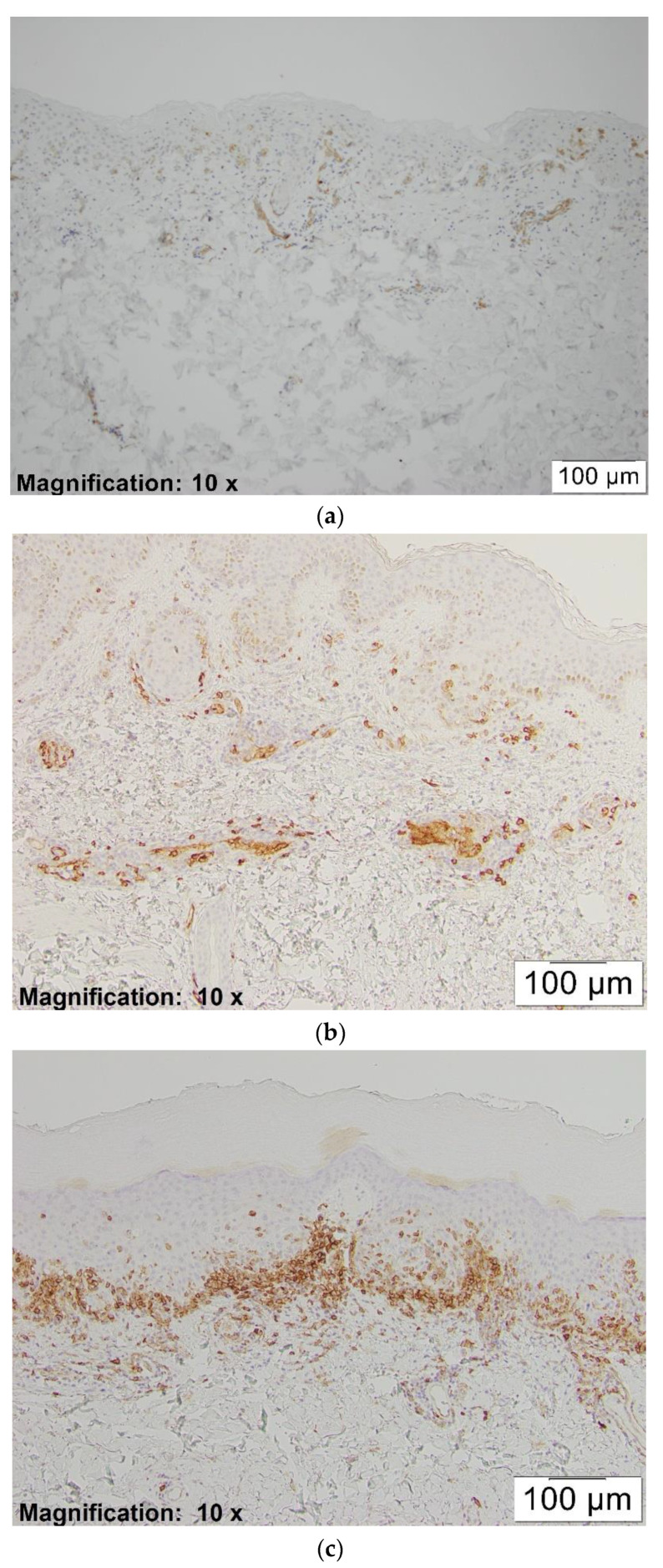
(**a**) CD123, ×10. This figure shows 1+ staining pattern of CD123 in drug induced erythema multiforme (EM). (**b**) CD123, ×10. This figure shows 2+ staining pattern of CD123 in other viral infection induced EM. (**c**) CD123, ×10. This figure shows 3+ staining pattern of CD123 in herpes simplex virus (HSV)-associated EM.

The positivity of CD123 was designated as dark-brown cytoplasmic staining of pDCs. Since these cells are not present in the skin under normal conditions, we assigned the cases as positive if strong or moderate staining of at least one plasmacytoid dendritic cell was identified. Subsequently, we assessed their density in the dermal inflammatory infiltrate by counting the pDCs and other inflammatory cells from a representative area at 20×. They were scored as: 1+ if the pDC percentage in the dermal inflammatory infiltrate was between 1–10%; 2+ if the percentage was between 11–50%; and 3+ if the percentage was more than 50% (see Figure 1a–c).

We evaluated epidermal involvement of pDCs and possible differences in staining pattern between etiologic categories. Chi-square test was performed and the *p*-value was calculated to assess the statistical significance of staining difference between virally induced (herpes and other viral induced) and non-virally induced (idiopathic, m. pneumonia and drug induced) cases of EM.

We also performed analysis to explore a possible relationship between the density of the staining and age, gender as well as severity of the disease (EM major vs. EM minor).

## 3. Results

We grouped the cases according to the etiologic category and percentage of the pDCs in the dermal inflammatory infiltrate (see Table 1).

**Figure 2:**
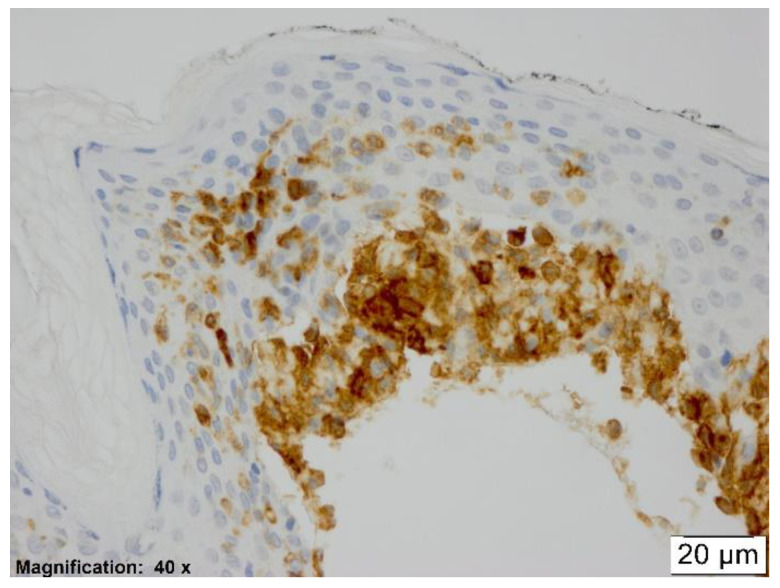
CD123, ×40. This figure shows epidermal plasmacytoid dendritic cells (pDCs) that have epithelioid and spindle shape.

In all etiologic groups of erythema multiforme, a significant amount of CD123 positive cells was observed within the dermal inflammatory infiltrate. Every case, no matter the underlying etiology, showed exocytosis of pDCs into the epidermis (see Figure 2).

**Figure 3:**
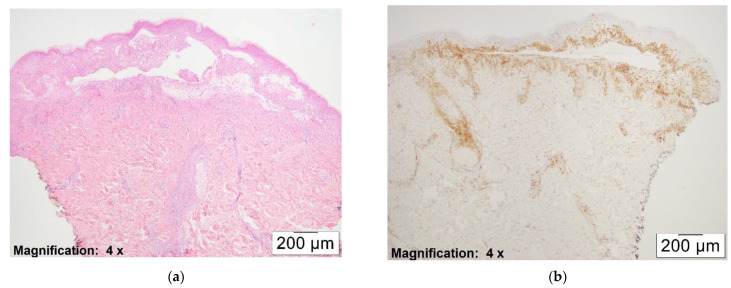
(**a**) H&E, ×4. This figure shows actively blistering mycoplasma pneumonia induced EM. (**b**) CD123, ×4. This figure shows dense inflammatory infiltrate with pDCs in the same case (3+ staining pattern).

Overall, the number of pDCs observed correlated with the extensiveness of the dermal infiltrate. Actively blistering areas with dense lymphocytic inflammation showed a higher density of pDCs (see Figure 3a,b)

**Figure 4:**
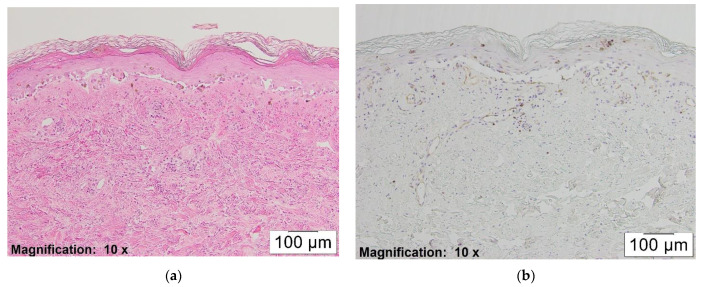
(**a**) H&E, ×10. This figure shows mycoplasma pneumonia induced EM. (**b**) CD123, ×4. This figure shows mild inflammatory infiltrate with pDCs in the same case (1+ staining pattern).

At the same time, partially or fully re-epithelialized older lesions with less lymphocytic inflammation revealed minimal staining with CD123 (see Figure 4a,b).

When we excluded the cases from the “unknown etiology” category and grouped the remaining cases as virally induced (herpes and other viral induced) versus non-virally induced EMs (idiopathic, m. pneumonia and drug induced), virally induced EMs showed a higher number of pDCs in the dermal infiltrate and were more likely to stain 2+ and 3+ (*p*-value < 0.05). In other words, a case that showed more than 10% pDC involvement was more likely to have a viral etiology (see Table 2).

In HAEM, all cases demonstrated more than 10% pDCs in the dermal inflammatory infiltrate, while in the other viral infectious category, 75% of the cases showed more than 10% involvement by pDCs. This observation might suggest that pDCs play a significant role in the pathogenesis of predominantly virally-induced EMs. It was also noted that a significant number of cases with unknown etiology stained 2+ or 3+ and hence had high number of pDCs in the dermal infiltrate. (see Table 1). This might suggest a previously unrecognized association with an antecedent virus in these cases, though we cannot prove this from the chart review.

In addition, in all cases, dermal pDCs were located particularly at the dermo-epidermal junction and around the superficial vessels. Interestingly, a higher number of pDCs was observed surrounding the vessels in close vicinity to the epidermis.

The pDCs detected appeared to be mostly rounded, epithelioid shaped, or occasionally spindly, dendritic shaped (see Figure 2)

Lastly, we did not observe any statistically significant association between the density of pDCs in the dermal infiltrate and age, gender, or severity of the disease (data not included).

## 4. Discussion

Similar to Herold et al. [5] and Amode et al. [10], we demonstrated the presence and hence presumed functional involvement of pDCs in the pathophysiology of erythema multiforme. In addition to their data, we observed pDCs to be located not only at the basal layer of epidermis but also at its upper parts. The most reasonable explanation for this fact lies in the discrete abilities of pDCs to regulate the inflammatory process and possibly the pathophysiology of erythema multiforme.

Although it has been discussed for decades, the pathophysiology of EM is still not well documented. Pathology of HAEM begins with the transportation of HSV DNA fragments to distant cutaneous parts by the mononuclear cells (PBMC) [9]. Upon the expression of viral genes by keratinocytes, HSV-specific CD4+ Th1 cells transiently infiltrate the lesion and produce IFN-γ to recruit additional inflammatory cells [9]. Interestingly, the main inflammatory response is carried on by the autoreactive T-cells [9]. This data suggests that HAEM might have an autoimmune component as well [9]. On the other hand, TNF-α, which is mainly produced by monocytes rather than activated T-cells, was shown to be a major cytokine in drug induced erythema multiforme (DIEM) [9]. These leading cell types and cytokine variations brought up HAEM and DIEM as mechanically distinct syndromes [9].

Due to the accumulated data on pDC involvement in wide variety of lichenoid reactions/interface dermatitis (LTR/IFD), it was suggested that there might be a common inflammatory signaling pathway underlying these diseases [11]. IFN-α produced by pDCs plays a key role in amplification of cytotoxic T-cells which is shown to be the final effector cell type in LTR/IFD [11]. Recently, the theory that suggests epidermal keratinocytes to be a primary initiator of the skin’s response to harmful insults has also become widely explored [12]. It is believed that regardless of the initiating factor, the immune reaction plays a key role in the skin’s response to a large array of injuries [12]. It was demonstrated that keratinocytes are central to the initiation and manifestation of immune-mediated skin injury [12]. These cells are able to recognize pathogen-associated molecular patterns (PAMPs) of microbial origin and danger-associated molecular patterns (DAMPs), such as toxins and other irritants [12]. Upon recognition of PAMPs and DAMPs through Toll-like receptors (TLRs), they produce cytokines and chemokines to recruit and activate dendritic cells (DCs) and plasmacytoid dendritic cells [12]. DCs and pDCs further recruit and activate T lymphocytes to produce pro-inflammatory cytokines, thus linking innate and adaptive immunity [12].

Our study demonstrated active involvement of pDCs in all cases of EM. This observation might suggest that the pathophysiology of etiologically different EMs might have a common pathway which involves pDCs. We propose that in EM, as with infectious pathogens or certain drugs, keratinocytes are activated through PAMPs and DAMPs and recruit pDCs for further inflammatory response as previously mentioned. On the other hand, we observed a significant difference in the number of pDCs in virally-induced EMs compared to non-virally-induced EMs (*p*-value < 0.05). This result might indicate that pDCs are more involved in virally induced EMs and may play additional roles. However, it should be noted that our results were only weakly significant due to the small number of cases. In order to make a stronger conclusion regarding this observation, a larger study would be necessary. In addition, a high number of cases with unknown etiology was also stained 2+ and 3+. It is sensible that HSV, which is identified as the most common culprit, might be a significant factor in this category and the staining pattern also supports this impression; however, it should be noted that we cannot prove this hypothesis based upon a chart review of these patients.

Depending on the anatomical location and the cytokine environment, pDCs may have both immunogenic and tolerogenic functions [13]. Schwab et al. divided pDCs into two subsets (pDC1 and pDC2) based on antigen expression by flow cytometry and functionality [14]. They report pDC1s to have tolerogenic functions and CD123^high^/CD58^low^ expression [14]. In contrast, the pDC2 was identified as an immunogenic cell with CD123^low^/CD58^high^ expression [14]. Immunogenic pDCs have been shown to be involved in the differentiation of naive CD4+ cells into effector Th1 cells, as well as the recruitment of activated T cells and memory differentiation [2,13]. Immunogenic pDCs are strong sensors of non-self nucleic acids derived from viruses through binding to Toll-like receptors (TLR) [13]. Upon the recognition of foreign nucleic acids, they produce massive amounts of type I IFN, and through this mechanism promote anti-viral effects [13]. On the other hand, tolerogenic pDCs have been demonstrated to be involved in the induction of central and peripheral tolerance [13]. Tolerogenic pDCs are able to induce anergy of CD4+ T cells as well as promote development of regulatory T cells [15]. The amount of pDCs we observed in our study necessitates a discussion of the significance of their role and function in EM.

In all cases of erythema multiforme we observed pDCs to be located in the upper dermis, as well as in the epidermis. Correlating epidermotropic abilities of pDCs with similar qualities of T- lymphocytes, one may conclude that epidermal pDCs are most likely those with immunogenic qualities. Unrelated to the underlying etiology of erythema multiforme, these cells most likely sense non-self or “masked” self-DNA and further promote immunologic response by recruiting effector T-cells and decreasing the viral spread. Nevertheless, we hypothesize that pDCs that we saw in the upper dermis might be of both immunogenic and tolerogenic types where dominance of one over the other might regulate the severity as well as the outcome. Hence the immunohistochemical analysis of CD123 expression may not clearly distinguish two populations of pDCs.

Immunogenic pDCs’ ability to recognize self-DNA plays an important role in the re-epithelialization as well [16]. Upon the formation of a skin wound, pDCs get activated by self-nucleic acids and initiate the early inflammatory response as well as increase the production of interleukin 6 (IL6), IL17, and IL22 [16]. These cytokines, especially IL22, are known to be important in epidermal regeneration as they promote keratinocyte proliferation and migration [16]. Similar to a skin wound, EM is characterized by epidermal destruction. We propose that immunogenic pDCs that are recruited to the skin during early inflammatory response, increase local cytokines and promote epidermal healing in EM. Therefore, we think their function might be crucial in the recovery process.

## 5. Conclusions

In conclusion, we highlighted the presence and involvement of pDCs in EM regardless of etiology. Although we do not know their role precisely, it is sensible to conclude that these cells play an important part in the pathophysiology of this entity and may have multiple different roles, depending upon the associated underlying disorder. Further studies are needed to clarify their importance in EM and by this means to shed light on the pathophysiology of this disease.

## Figures and Tables

**Table 1:** This table shows percentage of the pDCs in the dermal inflammatory infiltrate in relation to different etiologic categories as well as their epidermal involvement.

	Etiologic Category	Total Number of Biopsies	1+ (1–10%)	2+ (11–50%)	3+ (>50%)	Epidermal Involvement
Virally Induced EM ^1^	Herpes Associated EM ^1^	9	0/9 (0%)	4/9 (44%)	5/9 (56%)	9/9 (100%)
	Other Viral Induced EM ^1^	4	1/4 (25%)	2/4 (50%)	1/4 (25%)	4/4 (100%)
Non-Virally Induced EM ^1^	M. Pneumonia Induced EM ^1^	5	2/5 (40%)	1/5 (20%)	2/5 (40%)	5/5 (100%)
	Drug Induced EM ^1^	3	1/3 (33%)	1/3 (33%)	1/3 (33%)	3/3 (100%)
	Idiopathic EM ^1^	1	1/1 (100%)	0/1 (0%)	0/1 (0%)	1/1 (100%)
	Unknown Etiology	10	1/10 (10%)	3/10 (30%)	6/10 (60%)	10/10 (100%)

^1^ Erythema Multiforme.

**Table 2:** This table shows the results from the statistical analysis using the Pearson Chi-Square method.

	Number of Virally Induced EM 1 Cases	Number of Non-Viral Induced EM 1 Cases	Total
	% (*n*)	% (*n*)	
Low Density (1+)	20% (1/5)	80% (4/5)	5
High Density (2+/3+)	70% (12/17)	30% (5/17)	17
*p*-value ^2^	0.043		

^1^ Erythema Multiforme; ^2^ Pearson Chi-Square *p*-value.
